# The Odd Fellows' Convalescent Scheme

**Published:** 1888-10-27

**Authors:** 


### THE ODD FELLOWS' CONVALESCENT SCHEME.

The following are the rules relative to this scheme, which have lately been drawn up. They give many practical points which may be useful to others. The secretary is Mr. J. J. Robinson, Old Elvet, Durham: (1) That a convalescent home be established not earlier than May 1st, 1889, by and for the members of friendly societies. (2) That the necessary funds be provided by subscriptions from the lodges of friendly societies interested, who will receive tickets of admission in proportion to their subscription. (3) That each ticket admit a patient to the home for three weeks, and that the cost thereof for the first year of working be at the rate of 12*s.* per week. (4) That all subscriptions fall due in January of each year, all to be paid in advance, the financial year terminating on December 31st in each year. (5) That the scheme be put before the lodges in the county of Durham, at the earliest possible date, and that the subscriptions for the financial year 1889 be sought before December 31st, 1888, in order to enable sufficient accommodation to be provided. (6) That in the first instance the home be started in a suitable house, or houses be engaged for that purpose. (7) That the home be governed by a committee of management elected in or before January, 1889, consisting of an elected representative from each subscribing order. (8) That the session of officers, &c., and the arrangements of all working details as to the site, &c., be left to the committee of management to be elected by the subscribing lodges.

